# Patient-Reported Importance of Functional Benefit in Geographic Atrophy

**DOI:** 10.1001/jamaophthalmol.2025.3264

**Published:** 2025-09-25

**Authors:** Christiana Dinah, Jamie Enoch, Arevik Ghulakhszian, Mandeep Sekhon, Serena Salvatore, Gabriella DeSalvo, Praveen Kumar, Sanjiv Banerjee, Devaki Nayak, Winfried Amoaku, Marianne Shiew, Olayinka Osoba, David P. Crabb, Deanna J. Taylor

**Affiliations:** 1 Ophthalmology Department, Central Middlesex Hospital, London North West University Healthcare National Health Service Trust, London, United Kingdom; 2 Department of Brain Sciences, Imperial College London, London, England; 3 Department of Optometry and Visual Sciences, School of Health and Psychological Sciences, City St George’s, University of London, London, United Kingdom; 4 Population Health Research Institute, City St George’s, University of London, London, United Kingdom; 5 Ophthalmology Department, University of Bristol, Bristol, United Kingdom; 6 Ophthalmology Department, University Hospital Southampton National Health Service Trust, Southampton, United Kingdom; 7 Ophthalmology Department, University of Southampton, Southampton, United Kingdom; 8 Royal Berkshire Hospital National Health Service Trust, United Kingdom; 9 University Hospital of Wales, Cardiff, United Kingdom; 10 Shrewsbury and Telford National Health Service Trust, Shrewsbury, United Kingdom; 11 Research and Development, Keele University, Newcastle, United Kingdom; 12 University of Nottingham, Queens Medical Centre, Nottingham, United Kingdom; 13 Chelsea and Westminster National Health Service Foundation Trust, London, United Kingdom; 14 University Hospitals of Bristol & Weston, National Health Service Foundation Trust, Bristol, United Kingdom; 15 Torbay Hospital, South Devon National Health Service Foundation, Torquay, United Kingdom

## Abstract

**Importance:**

Intravitreal complement inhibitors injections (IVCIs) slowed progression of geographic atrophy (GA) lesions in several registration phase 3 trials although without benefit for prespecified secondary functional vision outcomes. Patient acceptability of these therapies needs further exploration.

**Objective:**

To quantify the acceptability of IVCI therapy to United Kingdom patients with GA, assuming vision outcome benefits are expected.

**Design, Setting, and Participants:**

This cross-sectional study took place at 9 geographically dispersed UK National Health Service centers from April 2023 to April 2024 among 153 participants with treatment-naive GA in at least 1 eye.

**Exposures:**

GA in at least 1 eye.

**Main Outcomes and Measures:**

Main outcomes were (1) acceptability of IVCI therapy based on completion of validated acceptability questionnaire. Participants were provided with a treatment information leaflet coproduced by a patients with lived experience of GA to inform them about the risks and benefits of IVCI for GA, assuming there were vision outcome benefits to this treatment and (2) response to the EuroQol 5-dimension with a vision bolt-on questionnaire to assess general health and vision-related quality of life. Spearman rank correlations and χ^2^ tests were used to explore associations between acceptability levels and specific ocular and sociodemographic characteristics.

**Results:**

A total of 153 participants were recruited (93 [60%] women; mean [SD] age, 82 [7]), 57 (38%) of whom had bilateral foveal involvement. Median (IQR) visual acuity with habitual correction in the better-seeing eye and in eyes where neither eye was better or worse was logMAR, 0.30 (0.14-0.54; approximate Snellen equivalent, 20/40) and 0.47 (0.14-0.84; approximate Snellen equivalent, 20/63), respectively. Among the 153 participants, 81 (53%; 95% CI, 45-61) reported IVCIs were very much or extremely acceptable under the theoretical scenarios provided. The proportion finding IVCIs acceptable rose to 82% (95% CI, 76-88) when including those who rated prospective treatment as moderately acceptable. Belief in the perceived effectiveness of the treatment (ρ, 0.52; 95% CI, 0.40-0.63; *P* < .001) and confidence in their ability to attend the eye clinic regularly (ρ, 0.51; 95% CI, 0.38-0.62; *P* < .001) correlated with overall acceptability.

**Conclusions and Relevance:**

IVCI therapy for GA may be acceptable to most UK patients with GA under the assumption that there are vision outcome benefits to this treatment. While current treatments do not result in vision outcome benefits, perceived effectiveness by patients was associated with acceptability, emphasizing the desire to quantify vision functional benefit concomitant with anatomical slowing of progression.

## Key Points

**Question:**

What is the perceived acceptability of intravitreal complement inhibitor (IVCI) therapy among patients with geographic atrophy (GA)?

**Findings:**

This cross-sectional study of 153 UK-based patients with GA, more than half found intravitreal injections for GA at least very acceptable, assuming treatment had vision functional benefits. Acceptability was influenced by participants’ belief that treatments would maintain vision longer compared with no treatment and confidence in their ability to regularly attend in-clinic injections.

**Meaning:**

For participants in this survey, IVCI therapy for GA was acceptable, assuming treatment had vision functional benefits, but careful counseling seems warranted to ensure patients understand the treatment’s anatomical and functional benefits and limitations.

## Teaser

This cross-sectional study evaluates the use of intravitreal complement inhibitor injections in the treatment of geographic atrophy lesions.

## Introduction

In the United Kingdom (UK), geographic atrophy (GA) accounts for approximately 26% of legal blindness, and globally approximately 5 million people have GA in at least 1 eye.^1^ Regular intravitreal injections are the standard of care for neovascular age-related macular degeneration (nAMD) and a common mode of delivery of therapies under investigation for treatments for GA. Dysregulation of the complement cascade has been implicated in the pathogenesis of GA.^2,3^ Recently, the US Food and Drug Administration approved 2 intravitreal complement inhibitors for GA treatment, pegcetacoplan and avacincaptad pegol. In randomized clinical trials, these treatments have been shown to slow down the anatomical progression of GA lesions.^4,5^ Unfortunately, no functional benefit was demonstrated in the prespecified analysis by 24 months. Furthermore, complement inhibitor treatments for GA may be associated with a 2- to 4-fold increased risk of nAMD in the treated eye compared to sham.^6^ These factors have contributed to lack of approval by the European Medicines Agency (EMA) and the Medicines and Healthcare products Regulatory Agency (MHRA).^7,8^

Amid debates regarding the safety and efficacy of these treatments, there has been limited focus on the perspectives of individuals with GA. Our mixed-methods pilot study^9^ in which we explored the views of UK-based patients with GA on the hypothetical acceptability of new intravitreal treatments suggested that the undefined benefits for functional vision and fear of developing nAMD were indeed considerable concerns. Additionally, patients were concerned about logistical burdens of regular hospital visits. However, despite potential burdens, approximately 60% found regular intravitreal therapy for GA acceptable. The main factor motivating acceptability in this pilot study was the high priority placed on the continuation of vision-specific activities, particularly for participants with worse self-reported health. While participants understood that functional benefit had not been demonstrated, they felt the reduction in anatomical growth of GA lesions would inevitably translate to the preservation of functional vision for longer.

Our pilot study^9^ was exploratory, involving 30 participants from 2 London-based sites, limiting its generalizability. In the current study, we seek to explore patient acceptability of intravitreal complement inhibitor therapies for GA in a larger, geographically diverse UK cohort. Both the pilot study and this present study were guided by the theoretical framework of acceptability (TFA), a framework used to guide research on acceptability of interventions. Acceptability is crucial in the design, evaluation and implementation of health care interventions.^10^ If an intervention is acceptable, patients are more likely to adhere to treatment recommendations and benefit from improved clinical outcomes. The TFA is underpinned by 7 component constructs, displayed with definitions and illustrative findings from the pilot study, in eAppendix 1 in Supplement 1. The TFA has been used to develop a generic, prevalidated quantitative questionnaire (TFA-Q) that can be adapted for assessing the acceptability of different health care interventions.^11^ In this study, we used the TFA-Q to measure patient acceptability of intravitreal complement inhibitor therapies for GA in a larger, geographically diverse UK cohort, and consider which aspects of intravitreal complement inhibitors injections (IVCIs) are more acceptable or unacceptable.

The principal objective of this study was to determine the proportion of patients with GA who judge IVCI therapy acceptable, using the TFA-Q. The secondary objectives were to determine correlations between demographic, general health, ocular factors, and overall acceptability score and correlations between specific items of the acceptability questionnaire and the overall acceptability score.

## Methods

### Ethical Considerations

The study received ethical approval from the Health Research Authority on and followed the Strengthening the Reporting of Observational Studies in Epidemiology (STROBE) reporting guideline. Written informed consent was obtained prior to data collection.

### Study Design

The research protocol was previously published in full.^12^ Briefly, this was a noninterventional, cross-sectional study conducted at 9 UK sites from April 2023 to April 2024. We approached 12 sites in the north, east, west, and south of the UK to provide geographical diversity, support generalizability of the study data and enable recruitment of the target sample size. Nine of these sites had the capacity to undertake the study and were included.

Participants with GA attending medical retina clinics for routine care provided written informed consent to participate in the study. No payments or incentives were provided for participation. They completed a demographic questionnaire and the EuroQoL 5-dimension (EQ-5D) questionnaire. We originally planned to use the National Eye Institute Visual Function Questionnaire 25 (NEI VFQ-25); however, participating sites judged its length burdensome for participants and a hindrance to recruitment. We therefore incorporated a vision bolt-on question into the EQ-5D, in line with previous studies.^13,14,15^ While the Functional Reading Independence Index^16^ is a patient-reported outcome measure used to assess reading abilities in individuals with geographic atrophy,^15^ given the focus of this study was treatment acceptability, rather than quality of life, and in the interest of not overburdening participants with too many questionnaires, we decided to include EQ-5D with a vision bolt-on question potentially to capture a more holistic picture of participants’ quality of life and to allow for greater comparability with other conditions. Best-corrected visual acuity with habitual correction (HCVA), optical coherence tomography, and fundus autofluorescence were performed as standard of care.

Subsequently, participants received a GA treatment information sheet that briefly explained GA, intravitreal complement inhibitor treatments in late-stage development, and the attributes of these treatments, including their risks and benefits (eAppendix 2 in Supplement 1). The information sheet (eAppendix 2 in Supplement 1) assumed vision functional benefits would accompany anatomical benefits of intravitreal complement inhibitor therapies for GA over time, even though the clinical trials demonstrated only anatomic benefits, with no prespecified functional benefits demonstrated vs sham. Following the delivery of this information, the TFA-Q was administered (eAppendix 3 in Supplement 1). We developed and validated this questionnaire using insights from our pilot mixed-methods study,^9^ our patient advisory group (including think-aloud testing with 10 participants with GA to ensure the included factors were the most relevant), expert clinical opinion, and a literature review.

### Patient and Public Involvement

Seven patient advisors with lived experience of GA have contributed to the Acceptability of Geographic Atrophy Injections (AGAIN) project since its inception in 2020, providing advice, particularly on study design and dissemination of findings. Advisors provided critical feedback on the draft acceptability questionnaire, assessing its clarity and relevance (face validity). Expert clinical review on the questionnaire was also sought.

### Participant Recruitment

Potentially eligible individuals were identified from GA patient databases at 9 geographically dispersed National Health Service trusts across the UK (in Wales, South England, North England, and West of England). Eligibility criteria are displayed in Table 1.

**Table 1.** Inclusion and Exclusion Criteria

Inclusion criteria	Exclusion criteria
Age ≥60 y	Macular disease in either eye due to causes other than AMD (eg, diabetic macular edema and Stargardt disease)
Diagnosis of GA in 1 or both eyes	Previously treated nAMD or retinal pigment epithelium rip in GA eye
GA lesion ≥0.5 disc area in size, as measured on fundus autofluorescence	Any concurrent ocular or intraocular condition that could contribute to central visual impairment
Visual acuity ≥24 in ETDRS letters (Snellen equivalent, 6/96) in either eye	Significant systemic disease or medication known to affect central visual function (eg, hydroxychloroquine toxicity)^17^
	Unable to understand consent process or participant information/questionnaires

Abbreviations: AMD, age-related macular degeneration; ETDRS, Early Treatment Diabetic Retinopathy Study; GA, geographic atrophy; nAMD, neovascular age-related macular degeneration.

### Statistical Analysis

Our pilot study found that IVCI injections were acceptable to 60% of participants (95% CI, 41-79) in a sample size of 30. With 164 participants, we calculated that acceptability can be estimated with a 95% confidence level and 7.5% width, for example expected to be between 52.5% and 67.5%.^9^

We analyzed the data using SPSS Statistics version 29 (IBM). We conducted descriptive analyses to calculate acceptability scores for different sociodemographic and GA characteristics. We analyzed the results by visual acuity subgroups based on the better-seeing eye, or in 1 eye among participants where the right eye was neither better or worse than the left eye, where a visual acuity difference of ±4 letters between eyes with visual acuity of logMAR 0.7 or greater (Snellen equivalent, ≥20/100) or ±9 in eyes with visual acuity less than logMAR 0.7 was deemed the same and a better-seeing eye was designated when the difference in visual acuity 5 letters or greater or 10 letters or greater between eyes with visual acuity of 20/100 or greater vs or 20/100 or less, respectively.^18^ In addition, we analyzed participants using logMAR 0.6 (20/80) as a visual acuity threshold. Correlations (Spearman rank) between acceptability scores, patient demographic characteristics, and clinical profiles (including previous experience of intravitreal injections for nAMD in the fellow eye) were determined. We planned to conduct multivariable regression analysis in the event of statistically significant correlations, to determine the simultaneous effects of multiple factors on patient acceptability.

We dichotomized acceptability into 2 categories: acceptable (ie, moderately, very, or extremely acceptable) and broadly unacceptable (ie, not at all or a little acceptable). Acceptability between different participant subgroups was compared using χ^2^ tests. EQ-5D scores between those who did and did not find injections acceptable were compared using the Mann-Whitney *U* test. We analyzed correlations (Spearman rank) between acceptability questionnaire items and overall acceptability on the TFA-Q, to explore how far the component constructs were associated with overall acceptability. Two-tailed *P* values less than .05 were considered statistically significant.

## Results

A total of 162 participants provided written consent. Nine of these were excluded from the data analysis due to missing primary outcome data. Participant characteristics for the remaining 153 and dichotomized acceptability levels for different subgroups, in terms of demographic and ocular characteristics, are displayed in Table 2.

**Table 2.** Subgroup Analyses of Acceptability by Self-Reported Demographic and Ocular Characteristics

Characteristic	No.^a^	Acceptable No./total No. (%)	Odds ratio (95% CI)	*P* value
Age, y				
≤80	152	51/62 (82)	1 (0.43-2.33)	.99
>80		74/90 (82)		
Sex				
Female	153	77/93 (83)	0.93 (0.40-2.16)	.46
Male		49/60 (82)		
Race^b^				
White	145	105/124 (85)	0.36 (0.13-1.01)	.05
Other^c^		14/21 (67)		
Highest education level				
High school/some college	146	88/103 (85)	0.55 (0.22-1.39)	.20
College degree		29/38 (76)		
Travel time to appointment				
≤30 min	146	57/68 (84)	0.88 (0.78-0.37)	.78
>30 min		64/78 (82)		
BCVA >0.6 in either eye				
No	145	25/30 (83)	0.98 (0.33-2.87)	.97
Yes		95/115 (83)		
BCVA >0.6 in both eyes				
No	145	91/104 (88)	0.35 (0.14-0.84)	.02
Yes		29/41 (71)		
BCVA in 2 eyes				
Has a better-seeing eye	145	84/101 (83)	1.30 (0.53-3.17)	.56
Has neither better- nor worse-seeing eye		35/44 (80)		
GA in 1 or both eyes				
1 Eye	153	57/67 (85)	0.76 (0.32-1.80)	.53
Both eyes		70/86 (81)		
GA foveal involvement				
Neither eye	153	22/28 (79)	1.58 (0.51-4.87)	.68
1 Eye		58/68 (85)		
Both eyes		46/57 (81)	1.14 (0.37-3.48)	
Multifocal GA				
Neither eye	150	56/72 (78)	2.09 (0.76-5.80)	.35
1 Eye		44/50 (88)		
Both eyes		23/28 (82)	1.31 (0.43-4.00)	
Previous intravitreal treatment for nAMD in the fellow eye				
No	153	74/93 (80)	1.67 (0.68-4.10)	.26
Yes		52/60 (87)		
No. of injections (for those previously treated)		22/25 (88)		.66
≤10	56^d^	22/25 (88)	0.71 (0.15-3.31)	.66
>10		26/31 (84)		
EQ-5D vision bolt-on score				
No/slight problems	143	53/62 (85)	0.75 (0.30-1.84)	.53
Moderate/severe/extreme problems		66/81 (81)		

Abbreviations: BCVA, best-corrected visual acuity; EQ-5D, EuroQoL 5-dimension; GA, geographic atrophy; nAMD, neovascular age-related macular degeneration z

^a^ Denominator varies due to missing data.

^b^ Race and ethnicity data were collected using a multiple-choice option and reported because there is evidence of underrepresentation of some races in ophthalmology research. As race and ethnicity can influence beliefs and cultural values, we felt it would be important to evaluate any association with preferences.

^c^ Other race and ethnicity groups included Black/Black African/Black British, Far East Asian, South Asian, and other (unspecified), consolidated owing to small numbers.

^d^ Including previously treated eyes when the number of previous injections was available.

The mean (SD) age of participants was 82 (7) years; 93 (60%) were women and 60 (40%) were men. Among participants for whom race was recorded, most (124/145 [85%]) were White, while 17 of 145 (11%) self-reported as South Asian and 4 as another race (including Black and unspecified, consolidated owing to small numbers). Almost all (145/150 [97%]) were retired, and 81 of 146 (55%) lived with a spouse or partner. Median (IQR) travel time to the hospital eye clinic was 35 (25-60) minutes.

Median HCVA in the better-seeing eye was logMAR 0.30 (IQR, 0.14-0.54; approximate Snellen equivalent, 20/40; 95% CI, 0.16-0.44) and 0.47 (IQR, 0.14-0.84; approximate Snellen equivalent, 20/63; 95% CI, 0.14-0.84) in 1 eye in participants where the right eye was neither better nor worse than the right eye. In addition, 41 of 145 (28%) of the cohort had HCVA better than 0.6 logMAR (Snellen equivalent, 20/80) in both eyes. More than half of patients (86/153 [56%]) had bilateral foveal involvement, while 28 (18%) had no foveal involvement in either eye. Seventy-eight of 150 patients (52%) had multifocal GA in 1 or both eyes, and 60 of 153 (39%) had previous injections to treat nAMD in their fellow eye.

Based on responses to the self-completed TFA-Q acceptability questionnaire, 81 of 153 participants (53%; 95% CI, 45 to 61) responded that the injections were very or extremely acceptable, and 45 of 153 (29%; 95 CI, 22 to 37) chose the scale midpoint of moderately acceptable, Thus 82% supported IVCI as moderately, very, or extremely acceptable (95% CI, 76-88), while 18% (n = 27; 95% CI, 12 to 24) indicated that the injections were not acceptable or only a little acceptable. Results from the acceptability questionnaire are displayed in Table 3 and the Figure. Before dichotomizing acceptability into broadly acceptable (responding moderately, very much or extremely acceptable) and not acceptable (not at all or a little acceptable), the only factor associated with acceptability was travel time to hospital, with longer travel time associated with lower acceptability of intravitreal injections (ρ, −0.18; 95% CI, −0.35 to −0.04; n = 146; *P* = .03). Higher EQ-5D scores (worse self-reported health) were associated with greater effort in attending the hospital eye clinic for monthly (ρ, 0.31; 95% CI, 0.16 to 0.44; n = 154, *P* < .001) and every other month (ρ, 0.26; 95% CI, 0.08 to 0.38; n = 153, *P* = .001) injections. When acceptability was dichotomized, patients with HCVA better than 0.6 logMAR in both eyes were less likely to find the injections acceptable (odds ratio [OR], 0.35; 95% CI, 0.14-0.84; *P* = .02).

**Table 3.** Responses to Theoretical Framework of Acceptability (TFA) Questionnaire, Including Associations Between Individual Acceptability Questions and Overall Acceptability Measure

Question	Respondents, No.	Response options	No. (%)	Correlation (95% CI) with question 10, overall acceptability item (*r*)^a^	*P* value of correlation coefficient
1. How comfortable (relaxed) do you feel about having these eye injections for geographic atrophy? (Affective attitude)	153	Very uncomfortable	24 (16)	0.44 (0.31 to 0.56)	<.001
		Little uncomfortable	45 (29)		
		No opinion	11 (7)		
		Comfortable	53 (35)		
		Very comfortable	20 (13)		
2. How much effort will it take for you to attend the clinic every month to receive these eye injections? (Burden)	153	No effort	46 (30)	−0.33 (−0.47 to −0.19)	<.001
		Little effort	59 (38)		
		No opinion	3 (2)		
		A lot of effort	30 (19)		
		Huge effort	15 (10)		
3. How much effort will it take for you to attend the clinic every 2 months to receive these eye injections? (Burden)	153	No effort	56 (37)	−0.30 (−0.43 to −0.14)	<.001
		Little effort	66 (43)		
		No opinion	6 (4)		
		A lot of effort	19 (12)		
		Huge effort	5 (3)		
4. How concerned are you about the increased risk of developing wet AMD with these injections? (Burden)	152	Extremely concerned	19 (13)	0.29 (0.14 to 0.43)	<.001
		Very concerned	28 (18)		
		Moderately concerned	39 (26)		
		Little concerned	41 (27)		
		Not concerned	25 (16)		
5. Attending the clinic regularly to receive these injections will be difficult for your relatives, family members, or caregivers. (Burden)	123	Not at all	57 (46)	−0.35 (−0.50 to −0.18)	<.001
		A little	38 (30)		
		No opinion	5 (4)		
		Very much	18 (15)		
		Extremely	5 (4)		
		Not applicable	30		
6. The injections will maintain my vision for longer. (Perceived effectiveness)	153	Strongly disagree	2 (1)	0.52 (0.40 to 0.63)	<.001
		Disagree	4 (3)		
		No opinion	40 (26)		
		Agree	76 (49)		
		Strongly agree	31 (20)		
7. It is clear to me how the injections will help maintain my vision. (Intervention coherence)	153	Strongly disagree	2 (1)	0.43 (0.29 to 0.55)	<.001
		Disagree	5 (3)		
		No opinion	25 (16)		
		Agree	92 (60)		
		Strongly agree	29 (19)		
8. How confident do you feel that you will be able to attend the clinic to receive the injections for the foreseeable future? (Self-efficacy)	153	Very unconfident	13 (8)	0.51 (0.38 to 0.62)	<.001
		Unconfident	13 (8)		
		No opinion	17 (11)		
		Confident	73 (47)		
		Very confident	37 (24)		
9. Attending the eye clinic to receive these injections will interfere with my other priorities. (Opportunity costs)	153	Strongly disagree	48 (31)	−0.30 (−0.44 to −0.15)	<.001
		Disagree	62 (41)		
		No opinion	16 (10)		
		Agree	19 (12)		
		Strongly agree	8 (5)		
10. Are the described eye injections for geographic atrophy acceptable to you?	153	Not at all	10 (7)	1.00 [Reference]	NA
		A little	17 (11)		
		Moderately	45 (29)		
		Very much	47 (31)		
		Extremely	34 (22)		

Abbreviations: AMD, age-related macular degeneration; NA, not applicable.

^a^ While some of the correlations are positive, and some negative, this simply reflects the fact that some questions were positively phrased and others negatively phrased.

**Figure.**
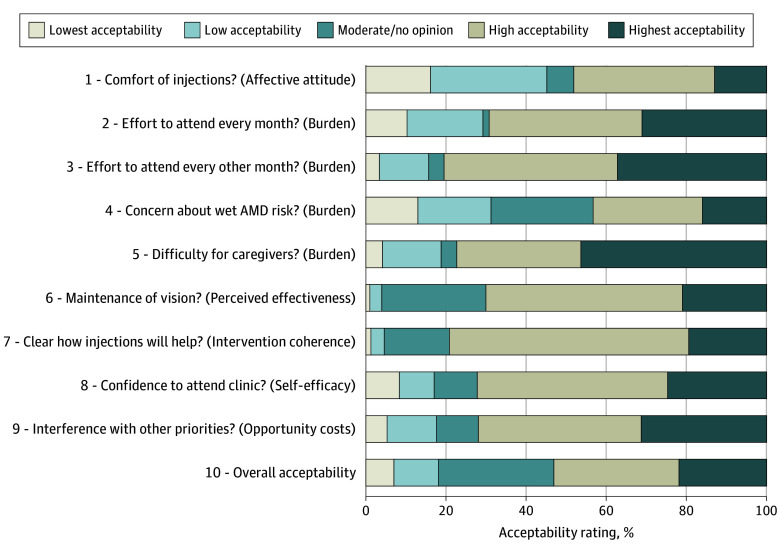
Simplified Responses to the Acceptability Questionnaire Response categories were simplified into least to most acceptable along a 5-point Likert scale, and reverse scores were converted to simplify pictorial representation.

There was no association between acceptability and EQ-5D score or other demographic factors (Table 4). However, there was a trend toward higher acceptability in White participants compared to participants of other races, although only of marginal statistical significance (shown in Table 2) and a smaller cohort of participants from other racial backgrounds.

**Table 4.** Association Between EuroQoL 5-Dimension (EQ-5D) Score and Acceptability

Acceptability of injections	No.	EQ-5D health index score, median (IQR)	Difference, median (95% CI)	*P* value
Unacceptable	27	0.75 (0.65 to 0.88)	−0.04 (−0.21 to 0.02)	.29
Acceptable	126	0.74 (0.60 to 0.84)		

When analyzing how the different acceptability subscales correlated with overall acceptability, all subscale items (questions 1-9) correlated with overall acceptability (question 10), as shown in Table 3. The questionnaire showed an adequate level of internal consistency (Cronbach α = .83). Using Spearman rank correlation, the strongest individual correlations with overall acceptability (question 10) were for question 6 (“the injections will maintain my vision for longer”: ρ, 0.52; 95% CI, 0.40-0.63), and for question 8 (“confidence to attend the clinic to receive the injections for the foreseeable future”: ρ, 0.51; 95% CI, 0.38-0.62). Considering the variance explained (by squaring the correlation coefficient), this suggests that 27% of variance in overall acceptability could be explained by the perceived effectiveness construct (question 6), and 26% by the self-efficacy construct (question 8).

## Discussion

In this UK-wide cross-sectional study, 53% of participants with GA who had been informed about the risks and benefits of IVCI therapy judged the prospect of such treatment for GA to be very or extremely acceptable under the assumption that vision functional benefits accompanied anatomical benefits of intravitreal complement inhibitor therapies for GA. As has been noted, these clinical trials showed only anatomic benefits (primary outcome of growth of GA from baseline to 12 or 18 months). No prespecified secondary outcomes evaluating functional vision functional benefits were superior to sham treatment.^4,5^ Including those who found the treatment moderately acceptable, the proportion of participants finding the treatment acceptable (extremely, very, or moderately) rose to 82% (126/153; 95% CI, 76-88).

The refusal to approve pegcetacoplan for GA by the MHRA and EMA may seem incongruous with the overall finding of our present study. However, further analysis showed that the strongest correlation with overall patient acceptability was perceived visual function effectiveness (“the injections will maintain my vision for longer”). While it may seem reasonable to expect that slowing the growth rate of geographic atrophy lesions will translate into functional benefit, this has not been demonstrated in any of the randomized trials^4,5^; despite multiple prespecified functional assessments such as BCVA, low luminance visual acuity (LLVA), reading speed, mean microperimetry sensitivity of prespecified locations, and NEI VFQ-25 scores.^17^ The failure of these randomized trials to demonstrate functional benefit despite anatomical benefit may be due to the phenotype of included participants. For example, nearly 90% of participants had subfoveal involvement on optical coherence tomography analysis at baseline in the Pegcetacoplan for the Treatment of Geographic Atrophy Secondary to Age-Related Macular Degeneration (OAKS and DERBY) trials,^5^ which might suggest a floor effect although the treated and control groups, on average, lost almost 10 letters over 2 years.^19^ Furthermore, more than 90% of participants in the Efficacy and Safety of Avacincaptad Pegol in Patients With Geographic Atrophy (GATHER2)^4^ study had foveal center-point sparing GA, yet, again, no evidence of functional benefit with BCVA or LLVA was identified compared with sham at 12 months. Functional benefit may occur over a longer time course, but the sham arm was discontinued in these trials after 2 years, making it difficult to determine if preservation of vision with complement inhibitor treatment compared with sham was occurring by 36 months in the open-label extension study for pegcetacoplan.^20^ Some authors have suggested that retinal pigment epithelium cells preserved with complement inhibitor therapy may be senescent and therefore unlikely to result in functional benefit.^21^ However, while the prespecified end point of mean sensitivity of microperimetry points did not reach significance at 24 months, a post hoc analysis of the OAKS study^22^ focused on perilesional and central macular microperimetry points, demonstrated reduced rate of visual function loss in pegcetacoplan-treated eyes at 24 months, perhaps warranting the need for additional clinical trials where this functional outcome is prespecified as a primary or principal secondary outcome.

Although only observed when both acceptability and HCVA variables were dichotomized, we found that people with better HCVA in both eyes were significantly less likely to find IVCI acceptable, with odds 65% lower than those with worse vision. This finding supports earlier reports that vision-related quality of life was correlated with BCVA in both eyes^23^ and aligns with qualitative insights from our pilot study,^9^ where participants with better BCVA were less accepting of the treatment, seeing limited value in such treatments while their everyday visual function was preserved. There is extensive evidence of difficulty reading and driving, recognizing faces, and visual impairment in dim lighting before BCVA is significantly reduced in some patients with GA.^24,25^ GA is heterogenous, with impact on functional vision depending on various factors such as focality, bilaterality, and exact location and portion of the central subfield occupied, rate and direction of growth within the macula and relative to the fovea. However, there is extensive evidence of difficulty reading and driving, recognizing faces, and visual impairment in dim lighting before BCVA is significantly reduced in some patients with GA.^23,24^. It is clear that the functional impact of GA varies between individuals, depending on factors such as focality, bilaterality, proximity to the fovea, and proportion of central subfield occupied. Improved understanding of the functional impact of the varied phenotypes and better tailored functional tests will be required to predict the functional impact of therapies for GA and inform counseling of patients.

Our current study demonstrated a correlation between greater travel time to hospital and lower treatment acceptability. This corroborates findings from our pilot study with 30 participants, where the logistical burden of regular clinic visits and concerns about adverse events were identified as key factors limiting acceptability of IVCI therapy for GA.^9^ Therefore, there may be value in considering care models via local, accessible community settings, such as shared-care models in collaboration with community optometry services.^26^ Furthermore, at the level of individual constructs, only in the case of the effort associated with injections EOM did at least 80% of participants choose the 2 most acceptable or positive of the 5 responses (ie, indicating little effort). This again points to logistical considerations as a key determinant of acceptability. Additionally, confidence in ability to attend the clinic to receive the injections for the foreseeable future showed one of the highest correlations with overall acceptability. The correlations between worse EQ-5D scores and increased effort to attend clinic reaffirm the importance of logistics and considerations for service design and delivery in GA, particularly for those who are in worse health or less mobile.

These results support a tailored, patient-centered approach to education and counseling, emphasizing shared decision-making between patient and clinician.^27^ In addition, social and economic factors often influence the functional impact of disease.^28^ Our findings suggest that attitudes toward treatment cannot be predicted solely using basic ocular profile or sociodemographic characteristics; but rather, beliefs about the functional impact of therapy underpin acceptability.

Our study’s multisite organization was a strength in recruiting a representative cohort of patients with GA in the UK, whose age, sex, race, and education level broadly represent the UK older adult population living with GA.^29^ In addition, the age, sex, proportion with bilateral GA, and visual acuity were similar to those of large, multicenter GA cohorts from the UK.^1^

### Limitations

Our study had several limitations that could affect the interpretation of the results. First, due to recruitment challenges, we only were able to recruit 153 participants against the 164 prespecified sample size.^12^ Underrecruitment may have reduced the statistical power to identify differences in acceptability levels based on ocular or demographic characteristics. Second, this study had the challenge of a priori defining a threshold for acceptability. In the absence of a generic, agreed threshold for acceptability, researchers recommend setting a prespecified threshold of acceptability before data collection, using previous research to provide a benchmark.^30^ Several recent articles use a figure of greater than 80% of acceptable or strongly acceptable responses as the threshold.^31,32,33,34^ Using this threshold, our study would suggest that the treatments are only acceptable when those who responded moderately acceptable are included; although conceivably, certain ambivalent participants may have chosen this response as the middle option of the 5-point range. In addition, the participants were told to assume there was a vision functional benefit to the treatment, even though such a benefit was not demonstrated within the prespecified secondary vision functional outcomes of clinical trials evaluating complement inhibitors for GA. Also, we used the vision bolt-on to the EQ-5D as a rapid measure of vision-related quality of life. While this score weakly correlated with foveal involvement status, it did not correlate with acceptability, and was likely too crude a tool to stage functional impact and its influence on acceptability. Furthermore, while the quantitative TFA questionnaire used in our study has been prevalidated,^11,35^ both the generic questionnaire and our adapted version of it have not undergone further psychometric testing.

An additional limitation concerns the lack of information provided to participants regarding the relatively rare risks of retinal vasculitis cases for those treated with pegcetacoplan post-marketing. This may have influenced acceptability, although it is impossible to know how far, given the low risk and association, typically, with the first injection.^36^

We did not collect data on characteristics of patients with GA who declined to participate in the study. As a noninterventional study taking place during standard-of-care clinics, most eligible participants agreed to participate once the NEI VFQ-25 was replaced with the vision bolt-on. Therefore, we are unable to determine whether potential participants that declined differed materially from those that participated.

## Conclusions

Overall, our cross-sectional study suggests that up to 82% of UK-based patients with GA could find IVCI therapy acceptable under the assumption that the therapy results in functional vision benefits. Critically, to date, clinical trials have only shown anatomic benefit as the primary outcome, whereas all prespecified functional vision outcomes, including BCVA, LLVA, reading speed, and patient-reported vision-related quality of life outcomes, were not shown to be superior in treatment compared with sham groups. Our results suggest that perception of functional benefit is key to patient acceptability when making decisions regarding intravitreal complement inhibitor therapy and highlight an urgent need for further research into functional phenotypes of GA and development of robust functional endpoints of clinical relevance to patients with GA. In health systems where IVCI or other emerging GA treatments become available for clinical use, a shared decision-making process that foregrounds patients’ unique preferences and circumstances remains essential.

## Notes

Supplement 1. eAppendix 1. The Theoretical Framework of Acceptability (TFA) component constructs, illustrated by relevant findings from the pilot study eAppendix 2. Information about GA and emerging treatments to be provided to study participants eAppendix 3. Acceptability questionnaire, based on generic Theoretical Framework of Acceptability questionnaire (Sekhon et al., 2022)

Supplement 2. Data sharing statement
