# Supplementary material for: Patient-Reported Importance of Functional Benefit in Geographic Atrophy
Source: JAMA Ophthalmol. 2025 Sep 25;143(11):916–24. doi: 10.1001/jamaophthalmol.2025.3264 (PMC12464851; doi:10.1001/jamaophthalmol.2025.3264)
Supplement: Supplement 1. — eAppendix 1. The Theoretical Framework of Acceptability (TFA) component constructs, illustrated by relevant findings from the pilot study eAppendix 2. Information about GA and emerging treatments to be provided to study participants eAppendix 3. Acceptability questionnaire, based on generic Theoretical Framework of Acceptability questionnaire (Sekhon et al., 2022) [file jamaophthalmol-e253264-s001.pdf]

Supplemental Online Content

Dinah C, Enoch J, Ghulakhszian A, et al. Patient-reported importance of functional benefit in geographic atrophy. JAMA Ophthalmol. Published online September 25, 2025. doi:10.1001/jamaophthalmol.2025.3264

eAppendix 1. The Theoretical Framework of Acceptability (TFA) component constructs, illustrated by relevant findings from the pilot study
eAppendix 2. Information about GA and emerging treatments to be provided to study participants
eAppendix 3. Acceptability questionnaire, based on generic Theoretical Framework of Acceptability questionnaire (Sekhon et al., 2022)

This supplemental material has been provided by the authors to give readers additional information about their work.

© 2025 Dinah C et al. JAMA Ophthalmology.

eAppendix 1. The Theoretical Framework of Acceptability (TFA) component constructs, illustrated by relevant findings from the pilot study

**Component construct in TFA**	**Definition within the TFA**	**Relevant findings per construct from pilot study**
Affective attitude	How an individual feels about the intervention	(+) Wish to delay further vision loss<br>(+) Good relationship with eye clinic staff<br>(-) Anxiety around intravitreal injections<br>(-) Discomfort of clip/speculum during injection procedure<br>(-) Long waiting times in clinic
Burden	The perceived amount of effort that is required to participate in the intervention	(+) Proximity to hospital<br>(+) Ease of travel to hospital<br>(-) Increased risk of wet AMD<br>(-) Regular travel to hospital<br>(-) Frequent treatment intervals<br>(-) Impacts on accompanying relatives/caregivers<br>(-) Concerns about side effects
Ethicality	The extent to which the intervention has a good fit with an individual's value system	(+) Belief that GA injections will help preserve independence<br>(-) Concerns about scarce NHS resources
Intervention coherence	The extent to which the participant understands the intervention and how it works (i.e. the 'face validity' of the intervention for the recipient)	(+) Clear understanding of anticipated treatment effects<br>(+) Understanding of the intravitreal injection process due to previous wet AMD treatment<br>(-) Confusion regarding improvement of vision<br>(-) Queries regarding treatment timeline<br>(?) Need for further information before treatment uptake

© 2025 Dinah C et al. JAMA Ophthalmology.

Opportunity costs	The extent to which benefits, profits or values must be given up to engage in the intervention	(+) Lack of time pressure<br>(+) Injections free at point of use for patients in the UK<br>(-) Waiting at eye clinic takes time away from valued activities
Perceived effectiveness	The extent to which the intervention is perceived as likely to achieve its purpose	(+) Anticipated benefits due to having vision for longer<br>(-) Belief that extra time with vision may not be worth it<br>(-) Belief that vision is currently good, therefore no perceived urgency for treatment<br>(-) Belief that vision-related quality of life has already deteriorated too much to benefit from treatment<br>(-) Difficulty of perceiving benefits of treatment first-hand
Self-efficacy	The participant's confidence that they can perform the behavior required to participate in the intervention	(+) Confidence to regularly attend eye clinic<br>(-) Concerns about feasibility of longer-term commitment to treatment

+ = Positive reflection of TFA construct

- = Negative reflection of TFA construct

? = Neutral reflection of TFA construct

© 2025 Dinah C et al. JAMA Ophthalmology.

eAppendix 2. Information about GA and emerging treatments to be provided to study participants

The GA treatment information sheet will summarise the following points in lay language:

- GA is the advanced form of dry age-related macular degeneration.
- It is slow to progress and starts outside the centre of vision in most people.
- It involves the centre of the vision within 5-7 years on average, causing difficulties seeing and performing vision-related tasks.
- Currently, there are two promising treatments being studied and likely to become available in clinics within the next 1-2 years.
- These treatments have all been found to be safe. Clinical trials are now being completed to determine how effective these treatments are in slowing down Geographic Atrophy.
- The key point to note is that these treatments will not cure, stop or reverse Geographic Atrophy. Instead, they will slow down the vision loss caused by Geographic Atrophy.
- It is estimated that these treatments can slow down vision loss by up to 20% at 1 year when delivered every month. As a concrete example: without treatment, a person could be five years away from having to stop driving because of Geographic Atrophy. However, if they were having the treatment, then they could potentially continue to drive for twelve months longer.
- For the treatment to keep working, it would involve lifelong, regular visits to the hospital for an injection into the eye. Currently, these visits could be every month or every other month.
- Every other month treatment slows down GA progression a little less than monthly injections.
- The treatments may cause temporary pain and discomfort in some cases, but severe side effects are rare. However, there is an increased risk of developing wet AMD with these treatments; up to 12% at 2 years with monthly treatment, and 7% with every other month treatment, compared to 3% without treatment.
- Injections in the eye can cause anxiety. However, people often feel much less anxious after having the first injection. Drops are used before these injections, in order to numb the eye and minimise any pain or discomfort.

© 2025 Dinah C et al. JAMA Ophthalmology.

eAppendix 3. Acceptability questionnaire, based on generic Theoretical Framework of Acceptability questionnaire (Sekhon et al., 2022)

1. How comfortable (relaxed) do you feel about having these eye injections for geographic atrophy?

O A) Very uncomfortable (1)

O B) A little uncomfortable (2)

O C) No opinion (3)

O D) Comfortable (4)

O E) Very comfortable (5)

2. How much effort will it take for you to attend the clinic **EVERY MONTH** to receive these eye injections?

O A) No effort at all (1)

O B) A little effort (2)

O C) No opinion (3)

O D) A lot of effort (4)

O E) Huge effort (5)

3. How much effort will it take for you to attend the clinic **EVERY TWO MONTHS** to receive these eye injections?

O A) No effort at all (1)

O B) A little effort (2)

O C) No opinion (3)

O D) A lot of effort (4)

O E) Huge effort (5)

© 2025 Dinah C et al. JAMA Ophthalmology.

4. How concerned are you about the increased risk of developing wet AMD with these injections?

- O A) Extremely concerned (1)
- O B) Very concerned (2)
- O C) Moderately concerned (3)
- O D) A little concerned (4)
- O E) Not concerned at all (5)

For each of the following statements [in Questions 5, 6, 7 and 9], please select/circle the option to indicate whether you strongly disagree, disagree, agree, strongly agree or you have no opinion.

5. Attending the clinic regularly to receive these injections will be difficult for your relatives/family members or caregivers

- O A) Not at all (1)
- O B) A little (2)
- O C) No opinion (3)
- O D) Very much (4)
- O E) Extremely (5)
- O F) Not applicable (N/A) (0)

© 2025 Dinah C et al. JAMA Ophthalmology.

6. The injections will maintain my vision for longer

- O A) Strongly disagree (1)
- O B) Disagree (2)
- O C) No opinion (3)
- O D) Agree (4)
- O E) Strongly agree (5)

7. It is clear to me how the injections will help maintain my vision

- O A) Strongly disagree (1)
- O B) Disagree (2)
- O C) No opinion (3)
- O D) Agree (4)
- O E) Strongly agree (5)

8. How confident do you feel that you will be able to attend the clinic to receive the injections for the foreseeable future?

- O A) Very unconfident (1)
- O B) Unconfident (2)
- O C) No opinion (3)
- O D) Confident (4)
- O E) Very confident (5)

© 2025 Dinah C et al. JAMA Ophthalmology.

9. Attending the eye clinic to receive these injections will interfere with my other priorities

- O A) Strongly disagree (1)
- O B) Disagree (2)
- O C) No opinion (3)
- O D) Agree (4)
- O E) Strongly agree (5)

10. Are the described eye injections for geographic atrophy acceptable to you?

- O A) Not at all (1)
- O B) A little (2)
- O C) Moderately (3)
- O D) Very much (4)
- O E) Extremely (5)

© 2025 Dinah C et al. JAMA Ophthalmology.
