# Supplementary material for: Patient-Reported Importance of Functional Benefit in Geographic Atrophy
Source: JAMA Ophthalmol. 2025 Sep 25;143(11):916–24. doi: 10.1001/jamaophthalmol.2025.3264 (PMC12464851; doi:10.1001/jamaophthalmol.2025.3264)
Supplement: Supplement 2. — Data sharing statement [file jamaophthalmol-e253264-s002.pdf]

Data Sharing Statement

Dinah. Patient-Reported Importance of Functional Benefit in Geographic Atrophy. *JAMA Ophthalmol*. Published September 25, 2025. doi:10.1001/jamaophthalmol.2025.3264

Data
Data available: No

Additional Information
Explanation for why data not available: In this study, we collected and analysed participants' clinical and demographic data that were sufficiently detailed to enable potential re-identification of participants. Therefore, the complete dataset cannot be made publicly available due to confidentiality concerns. Nonetheless, elements of the raw dataset (e.g. responses to the acceptability questionnaire) - with potentially identifying clinical/demographic information removed - are shareable. If this is of interest, please contact the corresponding author for further information.
